# Gut microbiota-derived ursodeoxycholic acid alleviates low birth weight-induced colonic inflammation by enhancing M2 macrophage polarization

**DOI:** 10.1186/s40168-022-01458-x

**Published:** 2023-01-31

**Authors:** Yu Pi, Yujun Wu, Xiangyu Zhang, Dongdong Lu, Dandan Han, Jiangchao Zhao, Xiaojiao Zheng, Shiyi Zhang, Hao Ye, Shuai Lian, Yu Bai, Zhenyu Wang, Shiyu Tao, Dongjiao Ni, Xinhua Zou, Wei Jia, Guolong Zhang, Defa Li, Junjun Wang

**Affiliations:** 1 State Key Laboratory of Animal Nutrition, College of Animal Science and Technology, China Agricultural University, Beijing, 100193 China; 2 Key Laboratory of Feed Biotechnology of the Ministry of Agriculture and Rural Affairs, Institute of Feed Research, Chinese Academy of Agricultural Sciences, Beijing, 100081 China; 3 State Key Laboratory of Biological Feed, Ministry of Agriculture and Rural Affairs, Boen Biotechnology Co. LTD, Ganzhou, 341000 China; 4 Department of Animal Science, Division of Agriculture, University of Arkansas, Fayetteville, AR 72701 USA; 5 Center for Translational Medicine, Shanghai Key Laboratory of Diabetes Mellitus and Shanghai Key Laboratory of Sleep Disordered Breathing, Shanghai Jiao Tong University Affiliated Sixth People’s Hospital, Shanghai, 200233 China; 6 Animal Nutrition Group, Wageningen University & Research, PO Box 338, Wageningen, 6700 AH The Netherlands; 7 University of Hawaii Cancer Center, Honolulu, HI 96813 USA; 8 School of Chinese Medicine, Hong Kong Baptist University, Kowloon Tong, Hong Kong, 999077 China; 9 Department of Animal and Food Sciences, Oklahoma State University, Stillwater, OK 74078 USA

**Keywords:** Low birth weight, Microbiome, Bile acids, Macrophage, Immunity

## Abstract

**Background:**

Low birth weight (LBW) is associated with intestinal inflammation and dysbiosis after birth. However, the underlying mechanism remains largely unknown.

**Objective:**

In the present study, we aimed to investigate the metabolism, therapeutic potential, and mechanisms of action of bile acids (BAs) in LBW-induced intestinal inflammation in a piglet model.

**Methods:**

The fecal microbiome and BA profile between LBW and normal birth weight (NBW) neonatal piglets were compared. Fecal microbiota transplantation (FMT) was employed to further confirm the linkage between microbial BA metabolism and intestinal inflammation. The therapeutic potential of ursodeoxycholic acid (UDCA), a highly differentially abundant BA between LBW and NBW piglets, in alleviating colonic inflammation was evaluated in both LBW piglets, an LBW-FMT mice model, and a DSS-induced colitis mouse model. The underlying cellular and molecular mechanisms by which UDCA suppresses intestinal inflammation were also investigated in both DSS-treated mice and a macrophage cell line. Microbiomes were analyzed by using 16S ribosomal RNA sequencing. Fecal and intestinal BA profiles were measured by using targeted BA metabolomics. Levels of farnesoid X receptor (FXR) were knocked down in J774A.1 cells with small interfering RNAs.

**Results:**

We show a significant difference in both the fecal microbiome and BA profiles between LBW and normal birth weight animals in a piglet model. Transplantation of the microbiota of LBW piglets to antibiotic-treated mice leads to intestinal inflammation. Importantly, oral administration of UDCA, a major BA diminished in the intestinal tract of LBW piglets, markedly alleviates intestinal inflammation in LBW piglets, an LBW-FMT mice model, and a mouse model of colitis by inducing M2 macrophage polarization. Mechanistically, UDCA reduces inflammatory cytokine production by engaging BA receptor FXR while suppressing NF-κB activation in macrophages.

**Conclusions:**

These findings establish a causal relationship between LBW-associated intestinal abnormalities and dysbiosis, suggesting that restoring intestinal health and postnatal maldevelopment of LBW infants may be achieved by targeting intestinal microbiota and BA metabolism.

Video Abstract

**Supplementary Information:**

The online version contains supplementary material available at 10.1186/s40168-022-01458-x.

## Introduction

Intrauterine growth restriction (IUGR), with approximately 30% prevalence in developing countries and approximately 8% in developed countries, is a significant public health concern worldwide [1]. IUGR impairs the growth and development of mammalian embryo/fetus or fetal organs during gestation, leading to low birth weight (LBW) [2], delayed growth, and permanent maldevelopment of infants during the postnatal period in humans, as well as livestock species [3]. Infants lower than 2500 g are defined as LBW by World Health Organization [4] and are often accompanied by digestive disorders such as necrotizing enterocolitis [3]. Of domestic livestock species, the pig exhibits the most IUGR occurrences and the pattern of growth retardation in the naturally occurring IUGR piglet is very similar to that which occurs naturally in the IUGR human neonate [5, 6], and it is an established animal model for studies of IUGR during prenatal and postnatal lives—that is, fetal programming and nutrient intervention [7–9]. In LBW piglets, the inflammatory response is heightened during the first 12 h after birth [10]. In addition, LBW also impairs the hindgut health associated with increased production of proinflammatory cytokines (e.g., IL-1β and TNF-α) and epithelial barrier dysfunction in pigs at the growing stage [11, 12].

The mammalian intestine, especially the large intestine, is colonized by a large number of bacteria (approximately 10^10^–10^12^ per gram of digesta) with high diversity (greater than 400 bacterial species) [13, 14]. One of many important functions carried out by the intestinal microbiome is the deconjugation of the primary bile acids (BAs) and subsequent biotransformation to the secondary BAs such as deoxycholic acid (DCA) and lithocholic acid (LCA) [15, 16]. Certain *Lactobacillus*, Erysipelotrichaceae, Lachnospiraceae, *Clostridium*, and *Bacteroides* encode the bile salt hydrolase (*bsh*) gene, while other *Lactobacillus*, Lachnospiraceae, Ruminococcaceae, Clostridiaceae, *Eubacterium*, and *Peptostreptococcus* carries the BA-inducible (*bai*) operon with the 7α-dehydroxylase activity [17–19]. The involvement of microbial BA dysmetabolism in inflammatory bowel disease (IBD) including ulcerative colitis and Crohn’s disease has been extensively studied [20]. Conjugated BA levels in the feces of IBD patients are higher than those in healthy individuals, while secondary BA levels are lower, due to an impairment of the deconjugation and transformation abilities of IBD-associated microbiomes [20].

However, a mechanistic understanding of these microbiome-BA-disease associations is lacking. Thus, the role of BA and its relationship with the intestinal microbiome in enteric diseases need to be elucidated and explored for potential therapeutic options [21]. The intestinal microbiota composition and fecal metabolome are known to be different between LBW and NBW piglets during early life [22, 23]. In addition, the total amount and synthesis of BAs in LBW infants are often reduced [24, 25]. BAs are well known to directly regulate the cells of innate immunity and act as a ligand of farnesoid X receptor (FXR) [26, 27]. Activation of FXR subsequently suppresses the release of IL-1β, IL-6, and TNF-α from LPS-primed macrophages [28, 29]. However, the microbial BA metabolism in LBW and the involvement of BAs in heightened intestinal inflammation in LBW remain largely unknown.

We hypothesized that the difference in the intestinal microbiota composition between LBW and NBW piglets alters the BA metabolism in the intestinal tract, resulting in heightened intestinal inflammation in LBW piglets. To test this hypothesis, targeted BA metabolomic profiling and microbiome analysis was employed to investigate the difference in the BA profile and microbiota composition in the intestines of LBW and NBW piglets in early life (two days after birth). Furthermore, both a DSS-induced colitis mouse model and a cell culture model were further employed to explore the role of ursodeoxycholic acid (UDCA), a secondary BA that is overly reduced in LBW piglets, in regulating intestinal inflammation and the potential underlying mechanisms involved. These studies will shed light on the dysregulation of the intestinal microbiota and BA metabolism in LBW, which will help elucidate the causes of the developmental defects associated with LBW and provide new approaches to the prevention and treatment of LBW-induced intestinal inflammation and postnatal maldevelopment.

## Materials and methods

### Piglets and fecal sample collection

A total of 14 litters of Duroc × (Landrace × Large White) piglets were naturally delivered from 14 multiparous sows (Landrace × Large White; 2-4 parities) after 113–114 days of gestation. Sows were fed a corn-soybean meal-based diet according to the NRC recommendations, with free access to water. The weights of newborn piglets were individually recorded immediately after birth, and one LBW piglet (0.75∼0.95 kg) and one NBW piglet (1.35∼1.55 kg) were obtained from each litter and housed individually in the same house. No cross-fostering was used in this study. None of the sows and piglets were administered antibiotics or any other drugs throughout the experiment. Fresh fecal samples were collected from the terminal rectum by a sterile cotton swab of 14 LBW and 14 NBW piglets on day 2 and then divided into two portions, with one flash-frozen in liquid nitrogen and then stored at −80°C until the microbiome and BA profiling analysis and the other portion used for fecal microbiota transplantation (FMT).

### Fecal microbiota transplantation

Fresh feces from both LBW and NBW piglets were diluted 5-fold individually in sterile PBS containing 15% glycerol (v/v), homogenized, aliquoted, and stored at −80 °C for future FMT to mice. A total of 16, 4-week-old male C57BL/6 SPF mice (Biotechnology Co., Ltd., Beijing, China) were treated with antibiotics (Meilun Bio., Dalian, China) (1 g/L streptomycin, 0.5 g/L ampicillin, 1 g/L gentamicin, and 0.5 g/L vancomycin) to deplete the intestinal microbiota as described [30]. The antibiotics were diluted in drinking water and replenished every other day. After 2 weeks of treatment, bacterial depletion in mice was confirmed using conventional bacterial culture on blood agar, McConkey agar, and Luria-Bertani (LB) broth (Beijing Land Bridge Technology Ltd., Beijing, China). Microbial DNA was further extracted from feces with QIAamp DNA Stool Mini Kit (Qiagen, Valencia, CA, USA), and total bacteria were quantified using quantitative PCR (qPCR) with universal primers for eubacteria as described [31]. For FMT, microbiota-depleted mice were orally inoculated with 0.2 mL of fecal microbial suspension from individual LBW or NBW donors once every other day for 4 weeks, with eight animals per treatment. Mice were weighed individually every 2 days, and colonic digesta and tissues were collected at the end of the study for further analyses.

### DSS-induced colitis in mice and ursodeoxycholic acid (UDCA) treatment

A total of 24, 8-week-old male C57BL/6 SPF mice were randomly divided into three groups, including a control group, a dextran sulfate sodium (DSS) group, and a DSS plus UDCA (Sigma-Aldrich, St Louis, MO, USA) enema group (50 mg/kg BW) (*n* = 8). Acute experimental colitis was induced with 3% DSS (36–50 kDa; MP Biomedicals, Santa Ana, CA, USA) in drinking water given ad libitum for 7 days. Body weight was measured daily throughout the experiment. Acute colitis was evaluated using a disease activity index (DAI) (Supplemental Table S1), colon length, and histological scoring system as previously described [32]. On day 8, mice were sacrificed, and the entire colon was removed for length measurement. A 2-cm segment of colon descendens was collected and fixed in 4% paraformaldehyde for processing for histopathological examinations.

### UDCA administration to LBW piglets

A total of 10 multiparous sows (Landrace × Large White; 2~4 parities) were fed a corn-soybean meal-based diet to meet the NRC recommendations, with free access to the water throughout the entire experiment. Ten litters of Duroc × (Landrace × Large White) piglets were spontaneously delivered from sows after 113∼114 days of gestation. The weight of the newborn piglet was recorded immediately after parturition, and 2 LBW piglets (0.75∼0.95 kg) were obtained from each litter and randomly allocated into either the control group with oral administration of 1 mL saline or the UDCA group with administration of 1 mL UDCA (50 mg/kg BW) daily (*n* = 10 in each group). All piglets were housed in the same environment. No cross-fostering was used in this study. None of the sows and piglets were administered antibiotics or any other drugs throughout the experiment. On day 8, six piglets were randomly selected from each group and sacrificed to collect the colonic tissue after weighing. Tissue samples were stored at −80°C until further analysis.

### Depletion of macrophages in mice

Macrophage depletion was performed by intraperitoneal injection of 0.2 mL clodronate liposomes (Liposoma BV, Amsterdam, The Netherlands) into mice 2 days before 3% DSS treatment, and also on day 1 and day 4 during DSS treatment as described [33]. Animals were randomly divided into five groups, including a control group, DSS group, DSS plus UDCA group, DSS plus macrophage depletion group, and DSS plus UDCA plus macrophage depletion group (*n* = 8 in each group). On day 8, mice were sacrificed for sample collection and further analysis as mentioned above.

### Fecal microbiota transplantation and ursodeoxycholic acid (UDCA) treatment

A total of 40, 4-week-old healthy male C57BL/6 SPF mice were selected. After 1 week adaptation period, 2 weeks of antibiotics intestinal microbiota were depleted, and 4 weeks of FMT of NBW or LBW fecal microbiota. Relevant experiment methods refer to the above description. Mice were divided into five groups, including an NBW-FMT group, an LBW-FMT group, an LBW-FMT + UDCA group, an LBW-FMT plus macrophage depletion group, and an LBW-FMT plus UDCA plus macrophage depletion group (*n* = 8 in each group). As for UDCA treatment, mice were orally administered 0.2 mL UDCA solution (50 mg/kg BW) every day. Macrophage depletion was performed 2 days before UDCA treatment, and on day 1 and day 4 during UDCA treatment. On day 8 of UDCA treatment, all the mice were sacrificed to collect the colonic tissue after weighing. Tissue samples were stored at −80°C until further analysis.

### Cell culture and treatments

Murine J774A.1 macrophages were cultured in DMEM (Invitrogen, Carlsbad, CA, USA) supplemented with 10% fetal bovine serum (Gibco, Carlsbad, CA, USA) and 1% penicillin/streptomycin (Invitrogen, Carlsbad, CA, USA) at 37°C with 5% CO_2_. Cells were seeded at 2 × 10^5^ cells/plate in a 6-well plate (Corning, Corning, NY, USA) overnight and then treated with 1 μg/mL LPS and 1 mM UDCA individually or in combination for another 24 h. The cells were harvested for RNA and protein extraction and subsequent RT-qPCR, RNA sequencing, Western blot, and flow cytometry analysis.

### RNA interference

Macrophages were transfected with double-stranded, FXR-specific small interfering RNA (forward 5′-CCA AGA ACG CCG UGU ACA ATT-3′ and reverse 5′-UUG UAC ACG GCG UUC UUGGTT-3′) or scrambled siRNA in 6-well plates using Lipofectamine® 3000 Reagent (Thermo Fisher Scientific, San Jose, CA, USA), followed by 1 mM UDCA treatment for 24 h. IL-1β concentrations in the supernatants were measured by ELISA. The cell samples were collected for gene expression of *IL-1β* and protein expressions of p65, p-p65, and FXR.

### Quantitative analysis of BAs

BAs in the feces and colonic digesta were profiled as previously described [34]. Briefly, a Waters ACQUITY UPLC coupled with a Waters XEVO TQ-S Mass Spectrometer with an ESI source controlled by MassLynx 4.1 software (Waters, Milford, MA, US) was used for all analyses. Chromatographic separations were performed with a Waters ACQUITY BEH C18 column (1.7 μm, 100 mm × 2.1 mm internal dimensions). Raw data obtained with a negative mode was analyzed using Waters TargetLynx Application Manager (version 4.1) to obtain calibration equations and concentrations of different BAs in each sample.

### Intestinal microbiome analysis

Total microbial genomic DNA in the feces and colonic digesta was extracted using QIAamp Fast DNA Stool Mini Kit (Qiagen Ltd., Hilden, Germany). The V3–V4 region of the 16S rRNA gene was amplified with the universal primers 341F (5′-ACTCCTACGGGAGGCAGCAG-3′) and 806R (5′-GGACTACHVGGGTWTCTAAT-3′). Raw sequences were analyzed using QIIME 2 (version 2020.2) [35]. Initial reads were quality filtered, denoised, assembled, and chimeric sequences were removed using Deblur [36], which generates unique amplicon sequence variants (ASVs) [36]. Only those ASVs with a minimum of two reads and present in more than two samples were retained. The phylogenetic tree was generated using the SEPP algorithm against the Silva 138 database under default settings [37]. The functional potential of the intestinal microbiota was predicted using PICRUSt2 [38]. All data were analyzed on the Majorbio I-Sanger Cloud Platform (https://cloud.majorbio.com/). Raw sequence reads were deposited into the NCBI Sequence Read Archive (SRA) database under accession number PRJNA646844 for the feces of piglets and PRJNA714049 for the colonic digesta of mice after FMT.

### Histopathological examinations

Colonic tissue samples were fixed in 4% paraformaldehyde for 24 h, followed by dehydration, embedding in paraffin, sectioning, and staining with hematoxylin and eosin or periodic acid-Schiff (PAS). Subsequent microscopic evaluations were made blindly by an experienced pathologist. The extent of tissue damage was scored as previously described [39]. The degree of epithelial loss on the intestinal surface, crypt destruction, and inflammatory cell infiltration were assessed and included in the histopathological examination (Supplemental Table 2).

### Flow cytometry analysis

Flow cytometry was conducted as previously reported [40]. Briefly, the colonic tissues were minced and incubated in Hank’s balanced salt solution (HBSS) containing 5 mM EDTA and 1 mM DTT at 37°C for 30 min to dissociate the epithelial cells. After filtration through a 100-μm cell strainer, the remaining pieces were incubated with an RPMI medium containing 0.05% collagenase D (Roche, Shanghai, China) and 0.05% DNase I (Roche, Shanghai, China) for 30 min with gentle shaking. As for J774A.1 cell samples, about 1 × 10^6^ cells per sample were collected after experimental treatment. Cell suspensions were then passed through a 70-μm cell strainer and collected for subsequent flow cytometry analysis. Lamina propria mononuclear cells (LPMCs) or J774A.1 cells was stained with the following antibodies: APC anti-mouse/human CD11b (Biolegend/M1/70), PE anti-mouse CD11c (Biolegend/N418), PE anti-mouse F4/80 (Biolegend/BM8), and APC anti-mouse CD206 (MMR) (Biolegend/C068C2). CD11b^+^CD11c^+^ cells were considered M1 macrophages, while F4/80^+^CD206^+^ cells were treated as M2 macrophages [40].

### RNA extraction and RT-qPCR

Total RNA was extracted from colonic tissues or J774A.1 cells using TRIzol (Takara Bio, Otsu, Japan), and 1 μg RNA was reverse-transcribed using PrimeScript® RT Reagent Kit with cDNA Eraser (Takara Bio, Otsu, Japan). RT-qPCR was performed with gene-specific primers (Supplemental Table 3) and an SYBR Green master mix on an ABI 7300 real-time PCR system (Applied Biosystems, Foster, CA, USA). Relative fold changes of gene expression were calculated using the cycle threshold (Ct) method and β-actin or GAPDH as a reference gene as previously described.

### RNA sequencing

For RNA sequencing, J774A.1 cells in 6-well plates were treated with UDCA at 1 mM with or without LPS (1 μg/mL) for 24 h. Total RNA was isolated using TRIzol, and RNA quality was assessed using a 2100 Expert Bioanalyzer (Agilent, Palo Alto, CA, USA) and sent for commercial library preparation and sequencing by Majorbio Biotech (Shanghai, China) on Illumina HiSeq 2000. Short sequence reads were analyzed on the Majorbio I-Sanger Cloud Platform (https://cloud.majorbio.com/). Based on GO annotation [41], differentially expressed genes (fold change ≥ 2 and FDR < 0.05) associated with biological processes, cellular components, and molecular functions were analyzed. Immune response-related genes were analyzed based on the KEGG pathway [42]. RNA sequencing data were deposited in NCBI’s Gene Expression Omnibus (GEO) under the accession number GSE174489.

### Enzyme-linked immunosorbent assay (ELISA)

The concentrations of IL-1β, TNF-α, and IL-6 in mouse colonic tissues and J774A.1 macrophage cell culture were measured using specific ELISA kits under catalog numbers 88-7013-88, 88-7324-88, and 88-7064-88 (Thermo Fisher Scientific, Waltham, MA, USA), respectively, according to the manufacturer’s instructions.

### Protein extraction and western blot analysis

Frozen mouse colonic tissues and J774A.1 cell pellets were ground using a mortar and pestle under liquid nitrogen and then lysed in RIPA buffer (150 mM NaCl, 1% Triton X-100, 0.5% sodium deoxycholate, 0.1% SDS, 50 mM Tris-HCl, pH 7.4) containing a cocktail of protease inhibitors (Thermo Fisher Scientific, Waltham, MA, USA). After centrifugation at 13,000×g for 10 min at 4°C, the protein concentration in the supernatant fluid was determined using a BCA Protein Assay Kit (Beyotime Biotechnology, Beijing, China). For Western blot, an equal amount of protein (30 μg) was run in SDS-PAGE together with pre-stained protein markers, transferred to polyvinylidene difluoride (PVDF) membranes (Millipore, Billerica, MA, USA), and blocked in 5% fat-free milk at room temperature in TTBS (20 mM Tris/150 mM NaCl, 0.1% Tween-20, pH 7.5) for 1 h. Membranes were incubated with primary antibodies, FXR (cat. no. 72105, 1:1000), p65 (cat. no. 6956, 1:1000), p-p65 (cat. no. 3033, 1:1000), p38 (cat. no. 8690, 1:1000), p-p38 (cat. no. 4511, 1:1000), ERK1/2 (cat. no. 4695, 1:1000), p-ERK1/2 (cat. no. 4370, 1:2000), JNK1/2 (cat. no. 9252, 1:1000), and p-JNK1/2 (cat. no. 4668, 1:1000) at 4 °C overnight with gentle rocking. All primary antibodies were purchased from Cell Signaling Technology (Danvers, MA, USA). After three washes in TTBS, the membranes were incubated with horseradish peroxidase-conjugated secondary antibodies (Goat anti-Rabbit IgG, Thermo Fisher Scientific, Waltham, MA, USA) at 1:5000 dilution for 1 h at room temperature. The signal was developed using Supersignal West Dura Extended Duration Substrate (Pierce, Rockford, IL, USA). Chemiluminescence was detected and quantified using Alpha Imager 2200 (Alpha Innotech, San Leandro, CA, USA) and normalized to the β-actin expression level in each sample.

### Statistical analysis

All statistical significance was assessed using one-way analysis of variance (ANOVA), followed by post hoc Tukey’s test for pairwise comparisons (SPSS 20 software, IBM, Armonk, NY, USA). All results were expressed as means ± SEM. Differences were considered statistically significant if *P* < 0.05. Orthogonal partial least squares-discriminant analysis (OPLS-DA) was performed to discriminate the intestinal BA profiles between treatment groups. Scatter plots were drawn using GraphPad Prism 6.0.

## Results

### Intestinal BA metabolism has differed between NBW and LBW piglets

**Fig. 1:**
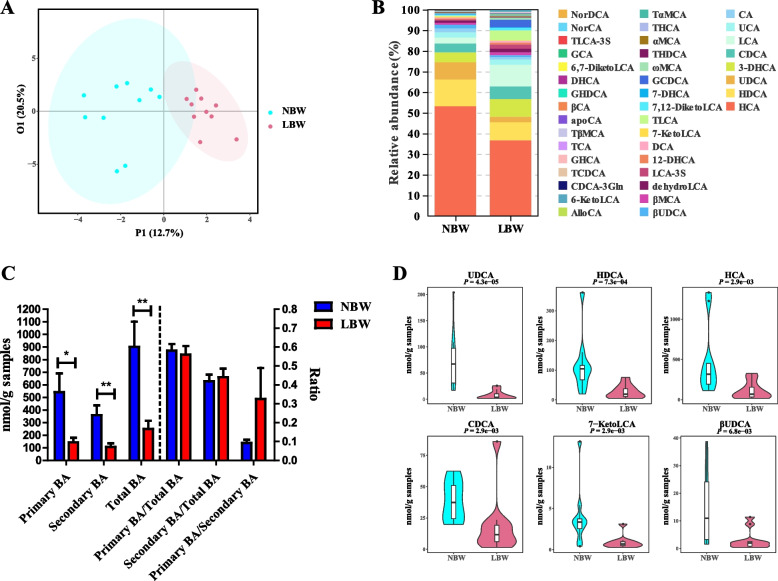
Fecal bile acid (BA) profiles of low birth weight (LBW) and normal birth weight (NBW) piglets 2 days after birth (*n* = 10). **A** OPLS-DA score plot of the fecal BA profiles. **B** Relative abundances of BAs in the feces of NBW and LBW piglets. **C** The concentration of primary BAs, secondary BAs, total BAs, and the ratio of primary BAs to total BAs, primary BAs to total BAs, and primary BAs to secondary BAs. **D** Violin plots of the six most differentially abundant fecal BAs. Data are presented as means ± SEM in panels **C** and **D**. *P < 0.05, **P < 0.01

To investigate whether BA metabolism is shifted in LBW piglets, targeted profiling of fecal BAs in day-2 NBW and LBW piglets was performed using UPLC-MS. Among a total of 40 BAs identified in the feces of piglets, the majority (75%) showed an increased level of NBW than LBW piglets (Supplemental Fig. 1A). OPLS-DA analysis showed a satisfactory Q^2^Y value of 0.511 and an R^2^Y value of 0.819 (Supplemental Fig. 1B), with a clear separation in the BA profile between NBW and LBW piglets (Fig. 1A). HCA, HDCA, and UDCA are the major BAs in the feces of piglets observed from the results of relative abundance (Fig. 1B). On the whole, the concentration of primary BAs, secondary BAs, and total BAs was significantly decreased in the LBW group, while the ratio of primary BAs to total BAs, secondary BAs to total BAs, and primary BAs to secondary BAs had no significant difference between NBW and LBW piglets (Fig. 1C). Thirteen highly differentially abundant BAs between two groups of piglets were further identified with a variable-importance-in-projection (VIP) value of > 1 (Supplemental Fig. 1C). LBW piglets showed a significant reduction of six BAs with the highest VIP values (UDCA, HDCA, HCA, CDCA, 7-Keto LCA, and βUDCA) compared to NBW piglets (*P* < 0.01) (Fig. 1D and Supplemental Fig. 1D). These results indicated that the BA metabolism in the intestinal tract differed between NBW and LBW piglets.

### FMT recapitulates LBW-characteristic intestinal microbiota and colonic inflammation in mice

**Fig. 2:**
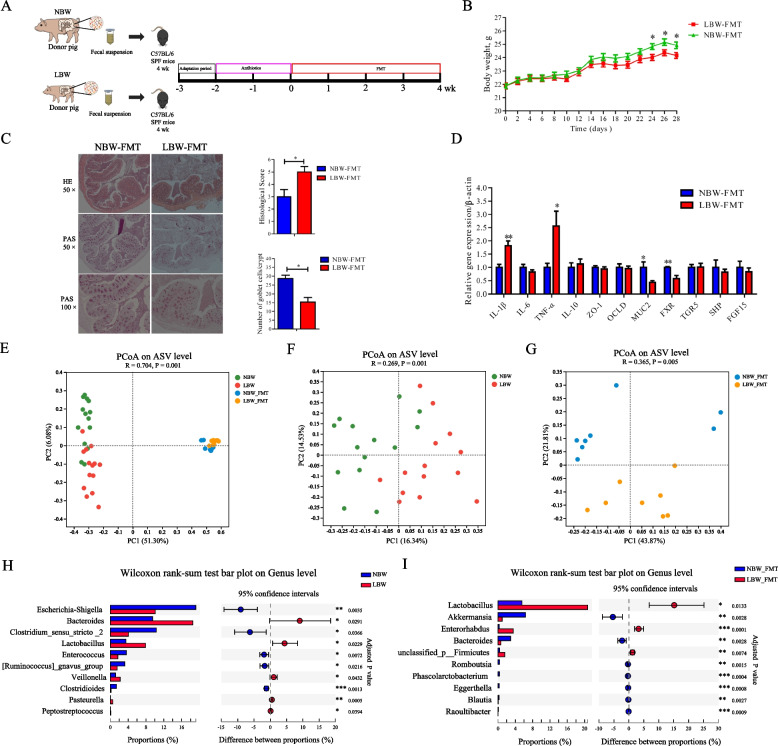
Effects of fecal microbiota transplantation (FMT) of NBW and LBW piglets to antibiotic-treated mice on gut microbial composition and intestinal health (*n* = 8). **A** Schematic outline of the experimental design. **B** Body weight changes of mice after FMT. **C** Representative H&E staining (50× magnification), PAS staining (50× and 100× magnification), histology score of the colonic sections, and the number of goblet cells of the mice after FMT. **D** The mRNA expression levels of genes related to inflammation, barrier function, and bile acid receptors in the colon of mice after FMT. Principal coordinates analysis (PCoA) plot of the microbiomes between the donor piglets and the recipient mice (**E**), and the fecal microbiomes of LBW and NBW piglets (**F**) or the colonic microbiomes of mice following FMT (**G**) based on the Bray-Curtis distance. Differential enrichment of bacterial genera between LBW and NBW groups (**H**) or between LBW-FMT and NBW-FMT groups (**I**) based on the Wilcoxon rank-sum test. Data are presented as means ± SEM. *P < 0.05, **P < 0.01. LBW-FMT, mice transplanted with the feces of LBW piglets; NBW-FMT, mice transplanted with the feces of NBW piglets

To examine whether the intestinal microbiota of LBW piglets is responsible for eliciting intestinal inflammation, FMT was performed with the fecal microbiota prepared from either LBW or NBW piglets to antibiotic-treated mice for the depletion of the intestinal microbiota (Fig. 2A). As expected, after oral administration of a cocktail of antibiotics for 2 weeks, mice showed an obvious decrease in culturable bacteria (Supplemental Fig. 2A) and the amount of bacterial DNA (Supplemental Fig. 2B) with a 4-log reduction in total bacteria in the feces (Supplemental Fig. 2C). FMT caused no differences in feed intake between LBW-FMT and NBW-FMT mice (*P* > 0.05) throughout the entire experiment (Supplemental Fig. 2D). However, the body weight of LBW-FMT mice was significantly reduced relative to that of NBW-FMT mice (*P* < 0.05) from day 24 after FMT (Fig. 2B). The colons of NBW-FMT mice were largely healthy, while LBW-FMT mice had fewer goblet cells and significantly higher histological scores (Fig. 2C). In addition, LBW-FMT mice showed a significant upregulation of the expressions of inflammatory cytokines (*IL-1β* and *TNF-α*) and downregulation of mucin-2 (*MUC2*) and BA receptor (*FXR*) in the colon (*P* < 0.05) (Fig. 2D).

To further examine the differences in the microbiota composition between NBW and LBW piglets and NBW-FMT and LBW-FMT mice, 16S rRNA gene sequencing was respectively performed with the fecal and colonic digesta bacterial DNA, showing a satisfactory sequencing depth (Supplemental Fig. 3A). The α-diversity indices such as ACE, Chao1, and Shannon index of the fecal microbiota of LBW piglets were markedly higher than those of NBW piglets (*P* < 0.05) (Supplemental Fig. 3B), while there were no significant changes in α-diversity between LBW-FMT and NBW-FMT mice (Supplemental Fig. 3C). On the whole, there were clear segregation of the microbiota structure of fecal samples between NBW and LBW piglets and colonic digesta samples between NBW-FMT and LBW-FMT mice based on the Bray-Curtis, weighted unifrac distance, and unweighted unifrac distance (ANOSIM, *P* < 0.05) (Fig. 2E and Supplemental Fig. 3D and G). In specific, clear segregation of the microbiota structure based on the Bray-Curtis (ANOSIM, *P* < 0.05), weighted unifrac distance (ANOSIM, *P* < 0.05) and unweighted unifrac distance (ANOSIM, *P* = 0.10) were observed between NBW and LBW piglets (Fig. 2F and Supplemental Fig. 3E and H), and also a clear separation of the microbiota in the Bray-Curtis, weighted unifrac, and unweighted unifrac distance were observed between the LBW-FMT and NBW-FMT groups (ANOSIM, *P* < 0.05) (Fig. 2G and Supplemental Figs. 3F and 4I). The microbiota was dominated by two phyla (i.e., Firmicutes and Bacteroidota) (Supplemental Fig. 4A) and three genera including *Escherichia-Shigella*, *Bacteroides*, and *Streptococcus* in LBW and NBW piglets while it was dominated by three genera including *Dubosiella*, norank_f_Muribaculaceae, and *Lactobacillus* in LBW-FMT and NBW-FMT mice (Supplemental Fig. 4B). LBW piglets showed a significant increase in the relative abundance of Bacteroidota and a decrease in the relative abundance of Proteobacteria, relative to the NBW piglets (*q* < 0.05) (Supplemental Fig. 4C). However, LBW-FMT mice showed a significant increase in the relative abundance of Actinobacteriota and a decrease in Desulfobacterota and Verrucomicrobiota as compared to NBW-FMT mice (Supplemental Fig. 4D). At the genus level, LBW piglets had significantly higher abundances of *Bacteroides*, *Lactobacillus,* and *Veillonella*, and lower abundances of *Escherichia-Shigella*, *Clostridium sensu stricto* 2, and *Enterococcus* than NBW piglets (*q* < 0.05) (Fig. 2H). However, LBW-FMT mice had higher abundances of *Lactobacillus* and *Enterorhabdus* and lower abundances of *Akkermansia* and *Bacteroides* (*q* < 0.05), relative to NBW-FMT mice (Fig. 2I). These results showed that the pattern of gut microbiota in mice after FMT was different from that of the donor piglets, suggesting that microbial function may be the key factor causing intestinal inflammation in LBW piglets.

### FMT recapitulates LBW-characteristic intestinal microbial BA-related function in mice

**Fig. 3:**
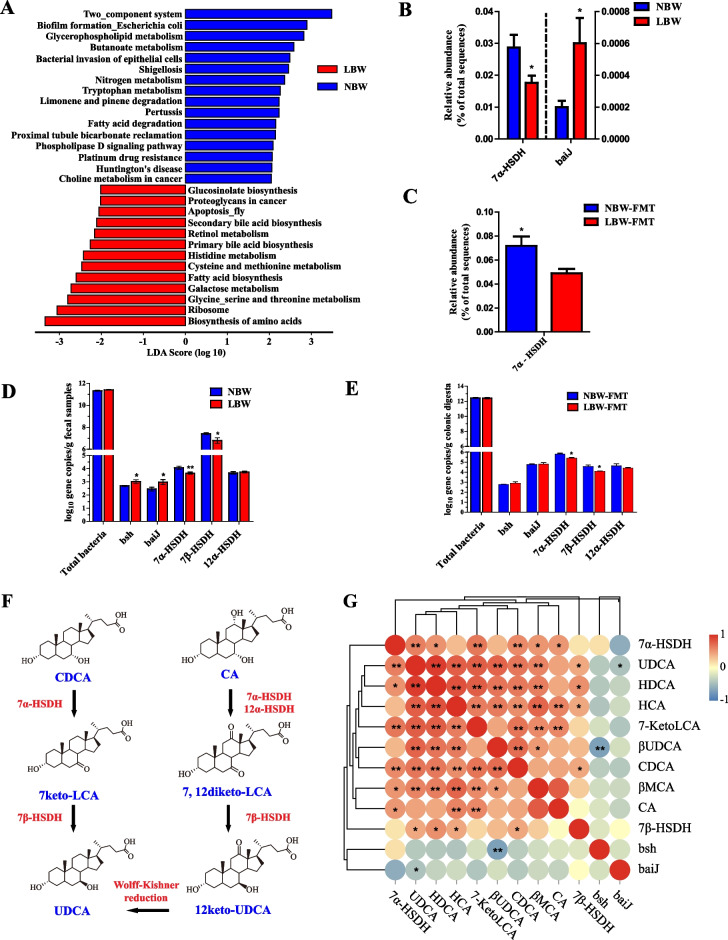
Microbial functional analysis in mice transplanted with the feces of NBW and LBW piglets (*n* = 8). **A** LEfSe analysis of the predicted microbial function based on PICRUSt2 between LBW and NBW groups. Differential enrichment of 7α-hydroxysteroid dehydrogenase (*7α-HSDH*) and BA 7-dehydroxylation (*baiJ*)-positive bacteria in the feces of LBW and NBW piglets (**B**) or colonic digesta of LBW-FMT and NBW-FMT mice (**C**) based on PICRUSt2 functional prediction. Gene copy numbers of *baiJ*, BA hydrolase (*bsh*), *7α-HSDH*, *7β-HSDH*, and *12α-HSDH* in feces of LBW and NBW piglets (**D**) or the colonic digesta of LBW-FMT and NBW-FMT mice (**E**) based on the qPCR analysis. **F** The synthesis pathway of UDCA by microbial biotransformation from CA and CDCA. **G** Spearman correlation between the gene copy numbers of individual BA-metabolizing genes and the concentrations of BAs in feces of LBW and NBW piglets. Data are presented as means ± SEM in panels **B**, **C**, **D**, and **E**. *P < 0.05, **P < 0.01. LBW-FMT, mice transplanted with the feces of LBW piglets; NBW-FMT, mice transplanted with the feces of NBW piglets

Functional prediction using PICRUSt2 revealed that the functions of primary BA biosynthesis and secondary BA biosynthesis were significantly altered by LBW (LDA > 2, *P* < 0.05) (Fig. 3A), the relative abundance of bacteria harboring the 7α-hydroxysteroid dehydrogenase (7α-*HSDH*) gene was significantly decreased, while BA 7-dehydroxylation (*baiJ*)-active bacteria were significantly increased in LBW piglets compared with NBW piglets (*P* < 0.05) (Fig. 3B). The relative abundance of bacteria harboring the *7α-HSDH* gene was also significantly reduced in LBW-FMT mice (*P* < 0.05) (Fig. 3C). Using qPCR, we further confirmed that *7α-HSDH* and *7β-HSDH* gene copy numbers were markedly decreased both in LBW piglets and LBW-FMT mice (*P* < 0.05) (Fig. 3D, E). It is noted that all BAs and microbial enzymes involved in the biosynthesis of UDCA from CDCA (Fig. 3F) were significantly reduced in LBW piglets (*P* < 0.05). Perhaps it is unsurprising that the gene copy numbers of *7α-HSDH* and *7β-HSDH* showed a significant positive correlation with fecal levels of UDCA (*P* < 0.05), while *baiJ* was negatively correlated with UDCA (*P* < 0.05) (Fig. 3G). These results suggested an obvious alteration in microbial BA metabolism in LBW piglets.

**Fig. 4:**
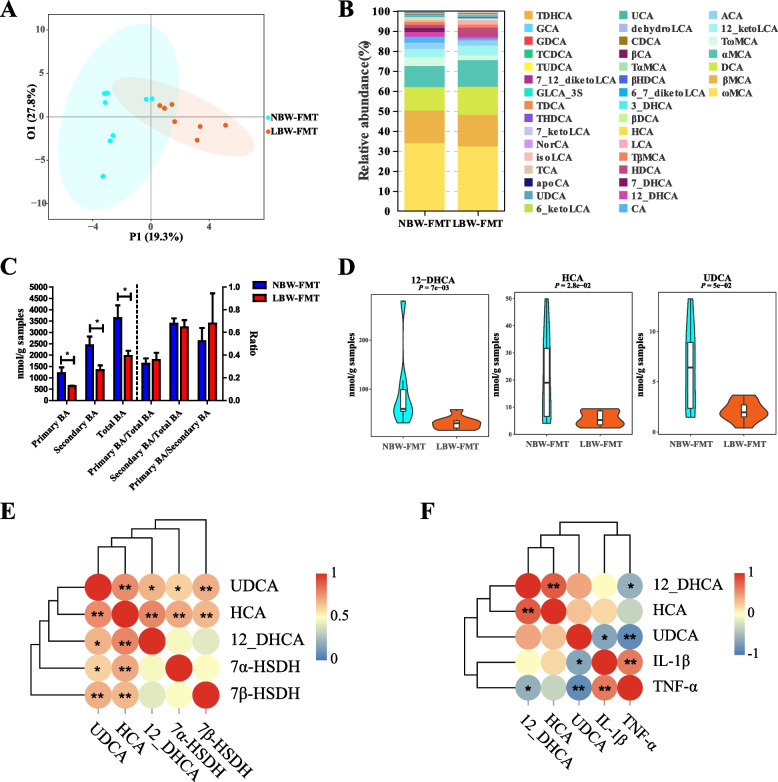
Colonic BA profiles of mice transplanted with the feces of LBW or NBW piglets (*n* = 8). **A** OPLS-DA score plot of the colonic BA profiles. **B** Relative abundances of BAs in the colonic digesta of NBW-FMT and LBW-FMT mice. **C** The concentration of primary BAs, secondary BAs, total BAs, and the ratio of primary BAs to total BAs, primary BAs to total BAs, and primary BAs to secondary BAs. **D** Violin plots of three differentially abundant colonic BAs. **E** Spearman correlation between the gene copy numbers of 7*α-HSDH* and *7β-HSDH* and the concentrations of three differentially abundant BAs in the colon. **F** Spearman correlation between the concentration of BAs and the gene expression of proinflammatory cytokines in the colon. Data are presented as means ± SEM in panels **C** and **D**. *P < 0.05, **P < 0.01. LBW-FMT, mice transplanted with the feces of LBW piglets; NBW-FMT, mice transplanted with the feces of NBW piglets

Targeted profiling of BAs revealed a reduction of most BAs in the colon of LBW-FMT mice (Supplemental Fig. 5A). An OPLS-DA model, as indicated by corresponding Q^2^Y and R^2^Y values, was suggested to be effective (Supplemental Fig. 5B) and showed a clear separation between NBW-FMT and LBW-FMT mice (Fig. 4A). ωMCA, βMCA, and DCA are the major BAs in the colon of mice after FMT observed from the results of relative abundance (Fig. 4B). On the whole, the concentration of primary BAs, secondary BAs, and total BAs was significantly decreased in the LBW-FMT group, and the ratio of primary BAs to total BAs, secondary BAs to total BAs, and primary BAs to secondary BAs had no significant difference between NBW-FMT and LBW-FMT piglets (Fig. 4C). A total of 19 highly differentially enriched BAs with a VIP value of > 1 were identified (Supplemental Fig. 5C). Relative to NBW-FMT mice, LBW-FMT mice showed significantly reduced concentrations of UDCA, HCA, and 12-DHCA (*P* < 0.05) (Fig. 4D and Supplemental Fig. 5D). Furthermore, the gene copy numbers of both *7α-HSDH* and *7β-HSDH* were significantly correlated with the UDCA concentrations in the colon (Fig. 4E). In addition, the gene expression levels of two proinflammatory cytokines (IL-1β and TNF-α) were significantly negatively correlated with the colonic concentrations of UDCA (Fig. 4F). These results indicated that the LBW phenotype can be recapitulated through interspecies FMT and the intestinal whole microbiome is responsible for BA metabolism and intestinal inflammation observed in LBW piglets.

### UDCA exerts anti-inflammatory and barrier-protective effects on both a mouse model of DSS-induced colitis and LBW piglets

**Fig. 5:**
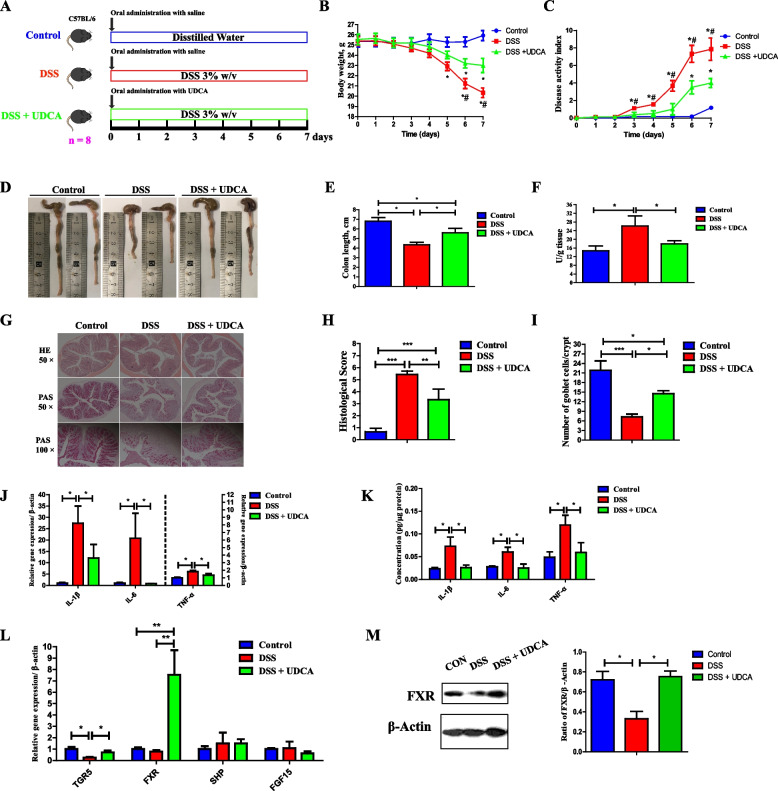
Amelioration of DSS-induced acute colitis in mice by oral administration of UDCA (*n* = 8). **A** Schematic outline of the experimental design. **B** Body weight changes of mice after FMT. **C** Disease activity index score. **D** Representative images of the colon. **E** Colon length. **F** Myeloperoxidase (MPO) concentrations in the colon. **G** Representative H&E staining (×50 magnification) and PAS staining (×50 and ×100 magnification) of the colonic sections. **H** Histology score of the colon. **I** The number of goblet cells. **J** The mRNA expression levels of inflammatory cytokines in the colon. **K** The protein levels of inflammatory cytokines in the colon. **L** The mRNA expression levels of BA receptors in the colon. **M** The protein levels of FXR in the colon. Data are presented as means ± SEM. *P < 0.05, **P < 0.01, *** *P* < 0.001; # *P* < 0.05, relative to the DSS + UDCA group

Given that UDCA is the most differentially abundant BA in LBW piglets (with the highest VIP value and the lowest *P*-value) showing a significant reduction in LBW-FMT mice and that such a reduction is associated with heightened colonic inflammation in both LBW piglets and LBW-FMT mice, we attempted to explore whether UDCA could directly prevent intestinal inflammation and barrier dysfunction. A mouse model of DSS-induced colitis was first employed with or without oral administration of UDCA at the beginning of a 7-day DSS treatment (Fig. 5A). UDCA significantly ameliorated DSS-induced colitis, as evidenced by markedly reduced weight loss (Fig. 5B), disease activity index score (Fig. 5C), and shortening of the colon length (Fig. 5D, E), and the myeloperoxidase (MPO) concentration in the colon (Fig. 5F). Consistently, UDCA administration led to significantly reduced inflammatory cell infiltration and epithelial layer destruction in DSS-treated mice (Fig. 5G, H). UDCA administration also significantly increased the number of goblet cells compared with the DSS group (Fig. 5I). In addition, UDCA suppressed both mRNA (Fig. 5J) and protein expression levels (Fig. 5K) of proinflammatory cytokines (IL-1β, IL-6, and TNF-α) in the colon. Additionally, UDCA upregulated both mRNA and protein expression levels of a BA receptor (FXR) as well as the mRNA expression of another BA receptor (*TGR5*) in the colon (*P* < 0.05) (Fig. 5L, M).

**Fig. 6:**
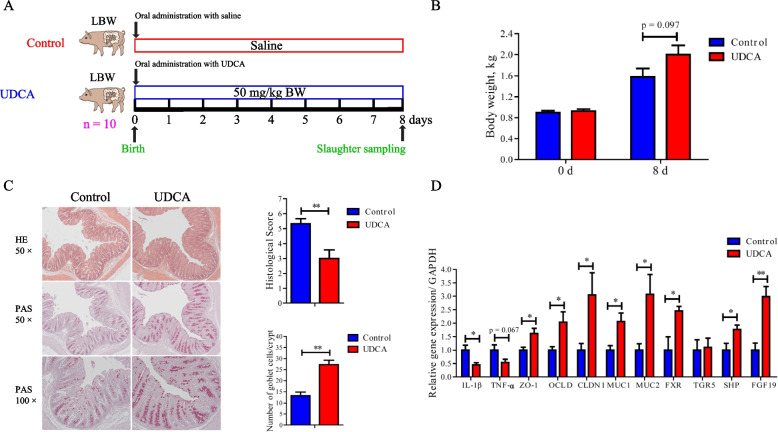
Alleviation of colonic inflammation by oral administration of UDCA in LBW piglets (*n* = 10). **A** Schematic outline of the experimental design. **B** Body weight before and 8 days after UDCA administration. **C** Representative H&E staining (×50 magnification), PAS staining (×50 and ×100 magnification), histology score of the colonic sections, and the number of goblet cells of LBW piglets receiving UDCA. **D** The mRNA expression levels of genes related to inflammation, barrier function, and bile acid receptors in the colon of LBW piglets receiving UDCA. Data are presented as means ± SEM. *P < 0.05, **P < 0.01

LBW piglets were further employed to confirm whether UDCA supplementation could reduce intestinal inflammation and improve gut health as observed in mice. UDCA (50 mg/kg) was orally administered to newborn LBW piglets daily for 7 days (Fig. 6A). Body weight on day 8 had a strong tendency to be higher than the control group (*P* = 0.097) (Fig. 6B). UDCA also clearly improved mucosal epithelial integrity and increased the frequency of goblet cells with a significantly reduced histological score in the colon, relative to the control (Fig. 6C). Moreover, similarly to what was observed in DSS-treated mice, UDCA significantly downregulated the mRNA expressions of IL-1β and TNF-α (Fig. 6D). The expressions of barrier function genes (*ZO-1*, *OCLD*, *CLDN1*, *MUC1*, and *MUC2*), as well as *FXR*, were also significantly increased in the colon of LBW piglets in response to UDCA treatment (Fig. 6D). These results strongly suggested that supplementation of UDCA may be of clinical utility to improve intestinal health and postnatal development of LBW infants.

### The anti-inflammatory effect of UDCA is achieved by inducing M2 polarization of macrophages

**Fig. 7:**
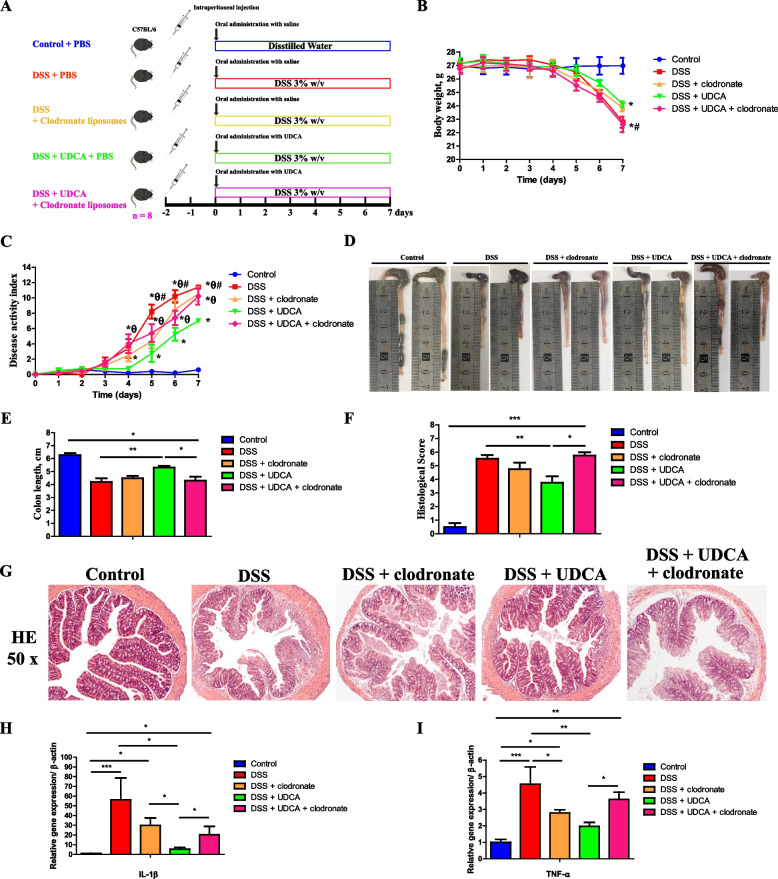
Effect of macrophage depletion on UDCA-mediated alleviation of intestinal inflammation in DSS-treated mice (*n* = 8). **A** Schematic outline of the experimental design. **B** Daily body weight changes. **C** Disease activity index score. **D** Representative images of the colon. **E** Colon length. **F** Histology score. **G** Representative H&E staining (×50 magnification) of the colon sections. The mRNA expression levels of two inflammatory cytokines, IL-1β (**H**) and TNF-α (**I**), in the colon. Data are presented as means ± SEM. *P < 0.05, **P < 0.01, *** *P* < 0.001; ^#^*P* < 0.05, relative to the DSS+UDCA group; ^θ^*P* < 0.05, relative to their respective clodronate treated groups

Macrophages are known to play a critical role in the progression of colitis [43]. To evaluate the role of macrophages in UDCA-mediated alleviation of colitis and intestinal inflammation, macrophages were depleted in mice through intraperitoneal injection of clodronate-containing liposomes 2 days before administration of DSS with or without UDCA intervention (Fig. 7A). Macrophage depletion significantly abrogated alleviation of DSS-induced colitis by UDCA, as evidenced by the body weight change (Fig. 7B), disease score (Fig. 7C), colon length shortening (Fig. 7D, E), colonic epithelial histology score (Fig. 7F), and inflammatory cell infiltration (Fig. 7G). In addition, macrophage depletion substantially abrogated UDCA-mediated suppression of IL-1β (Fig. 7H) and TNF-α (Fig. 7I) in the colon of DSS-treated mice.

**Fig. 8:**
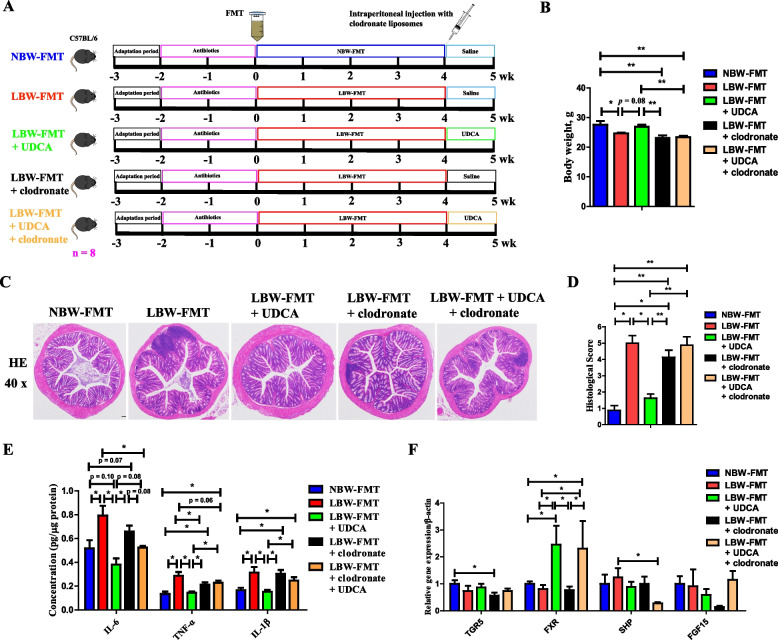
Effect of macrophage depletion on UDCA-mediated alleviation of intestinal inflammation in LBW-FMT mice (*n* = 8). **A** Schematic outline of the experimental design. **B** Body weight changes. **C** Representative H&E staining (×40 magnification) of the colon sections. **D** Histology score. **E** The mRNA expression levels of proinflammatory cytokines (IL-6, TNF-α, and IL-1β) and **F** bile acid receptors in the colon. Data are presented as means ± SEM. *P < 0.05, **P < 0.01. LBW-FMT, mice transplanted with the feces of LBW piglets; NBW-FMT, mice transplanted with the feces of NBW piglets

To further confirm the role of the macrophage in UDCA-mediated alleviation of LBW-induced intestinal inflammation, an LBW-FMT mouse model was used and macrophages were depleted in mice through intraperitoneal injection of clodronate-containing liposomes during UDCA intervention (Fig. 8A). Macrophage depletion significantly abrogated the alleviation of LBW-induced body weight loss (Fig. 8B) and colonic epithelial histology score (Fig. 8C, D) by UDCA. In addition, macrophage depletion substantially abrogated UDCA-mediated suppression of IL-1β, IL-6, and TNF-α (Fig. 8E) in the colon of LBW-FMT mice. Moreover, the FXR expression was significantly activated by UDCA intervention (Fig. 8F). These results indicated that UDCA exerts its anti-inflammatory effect in the colon mainly through the involvement of macrophages.

**Fig. 9:**
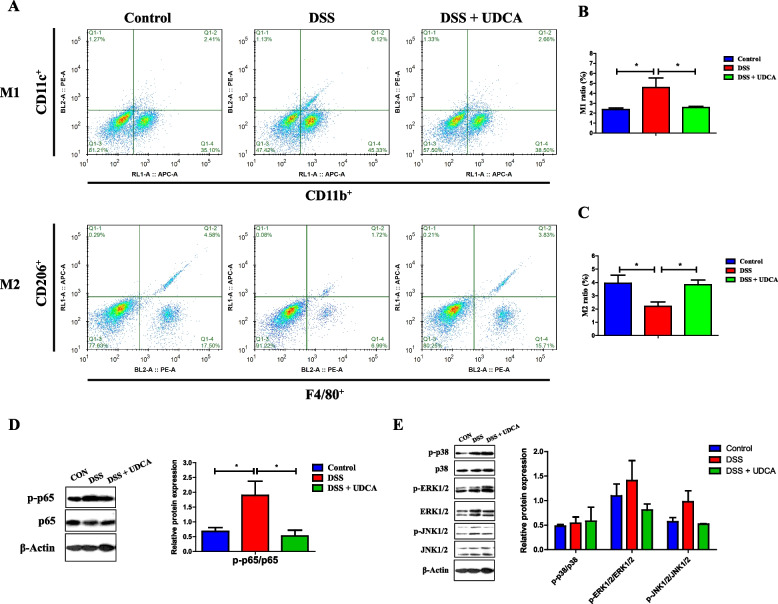
Effect of UDCA on macrophage polarization and NF-κB inhibition in DSS-treated mice (*n* = 8). Shown are representative flow cytometry plots (**A**) as well as the prevalence of M1 macrophages (CD11b^+^CD11c^+^) (**B**) and M2 macrophages (F4/80^+^CD206^+^) (**C**) in the colonic homogenate of DSS-treated mice in response to UDCA. **D** Decreased phosphorylation of p65 NF-κB in the colon of DSS-treated mice in response to UDCA. **E** Phosphorylation of p38, ERK1/2, and JNK1/2 in the colon of DSS-treated mice in response to UDCA. Data are presented as means ± SEM. *P < 0.05

Macrophages are polarized to become inflammatory M1 or anti-inflammatory M2 cells in response to the tissue microenvironment [44]. To study the impact of UDCA on macrophage polarization in intestinal inflammation, flow cytometry was performed with LPMCs prepared from the colon of DSS-treated mice receiving with or without UDCA. As expected, the percentage of M1 macrophages (CD11b^+^CD11c^+^) was increased and M2 macrophages (F4/80^+^CD206^+^) decreased in response to DSS treatment, while UDCA administration reversed the trend (Fig. 9A–C), suggesting that UDCA exerts an anti-inflammatory effect at least in part through regulation of macrophage polarization.

### The anti-inflammatory effect of UDCA is mediated in part through FXR and NF-κB suppression

NF-κB and MAPK signaling are key pathways involved in proinflammatory cytokine production. The role of UDCA in the activation of NF-κB and MAPK pathways was first examined in the colon of DSS-treated mice. DSS-induced phosphorylation of p65 NF-κB was significantly decreased by UDCA administration (Fig. 9D). However, no significant difference was observed in MAPK (p38, JNK, and ERK) signaling pathways in response to DSS or UDCA (Fig. 9E).

Murine J774A.1 macrophage cells were further employed to study the underlying mechanisms of UDCA-mediated anti-inflammatory response. While it did not affect cytokine gene expression in untreated cells, UDCA dose-dependently suppressed LPS-induced expressions of inflammatory cytokines (IL-1β, IL-6, and TNF-α) after 24-h treatment (data not shown). While it appeared to amplify LPS-induced inflammatory response at 6 and 12 h, UDCA markedly suppressed inflammatory cytokine expressions at 24 h (Supplemental Fig. 6A) and significantly increased the percentage of M2 macrophages (F4/80^+^CD206^+^) (Supplemental Fig. 6B and C).

**Fig. 10.**
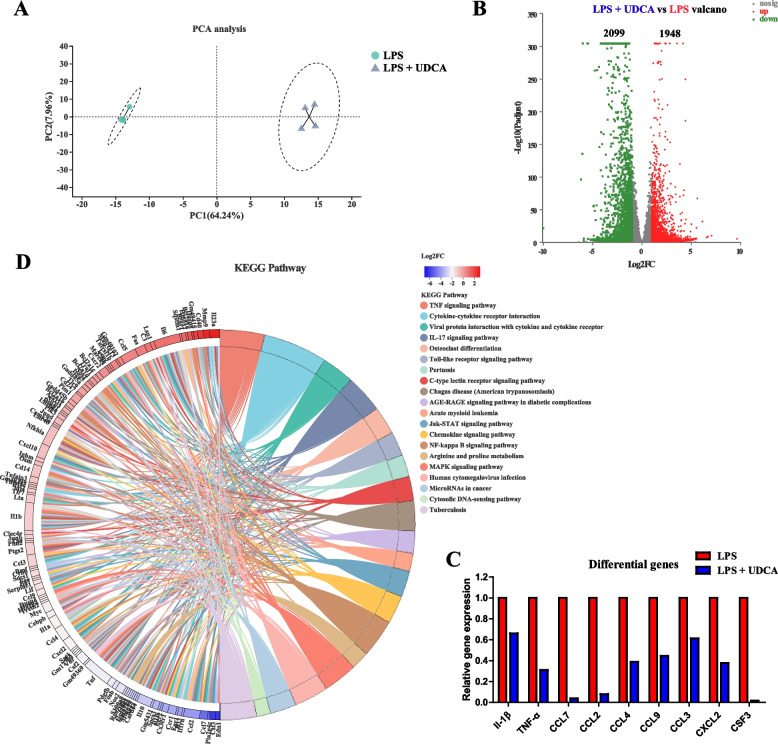
Transcriptional profiling of LPS-treated J774A.1 cells in the presence or absence of UDCA (*n* = 4). **A** PCA plot of the transcriptional profiles between the two groups. **B** Volcano plot of significantly differentially expressed genes between the two groups. The differentially expressed genes included only those showing a > 2-fold difference and FDR < 0.05. **C** Inhibition of a panel of LPS-induced proinflammatory cytokine and chemokine gene expression in the presence of UDCA. **D** Differential enrichment of KEGG pathways in LPS-stimulated cells following UDCA treatment

To understand the transcriptomic changes of LPS-stimulated J774A.1 macrophages in response to UDCA, RNA-seq was performed and revealed an obvious shift in the transcriptomic profile (Fig. 10A). UDCA caused a significant upregulation of 1948 genes and a significant downregulation of 2099 genes in LPS-stimulated J774A.1 cells (Fig. 10B). For example, UDCA suppressed LPS-mediated induction of a panel of proinflammatory cytokine and chemokine genes such as *IL-1β*, *TNF-α*, *CCL7*, *CCL2*, *CCL4*, *CCL9*, *CCL3*, *CXCL2*, and *CSF3* (Fig. 10C). KEGG pathway analysis further revealed differential downregulation of multiple pathways in LPS-treated J774A.1 cells in response to UDCA. Some of the pathways included TNF signaling pathway, cytokine-cytokine receptor interaction, Toll-like receptor signaling pathway, and NF-κB signaling pathway (Fig. 10D). These results further confirmed the anti-inflammatory functions of UDCA.

**Fig. 11:**
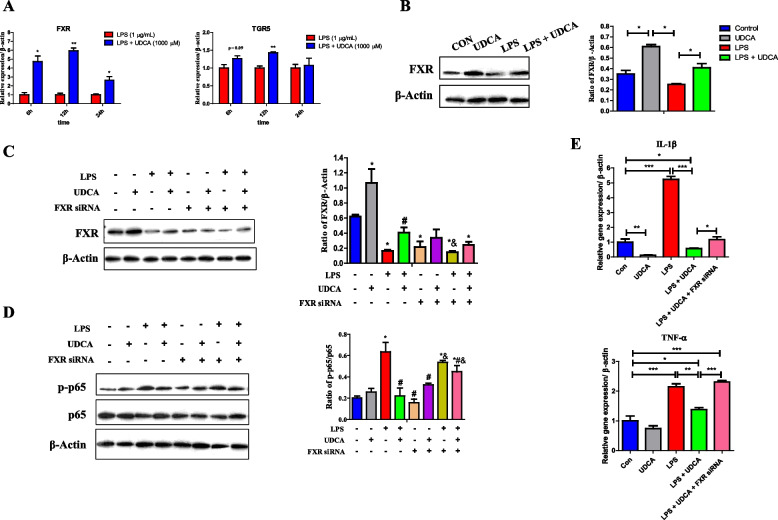
Involvement of FXR in UDCA-mediated anti-inflammatory effects in LPS-treated J774A.1 cells (*n* = 3). Shown are the expressions of *FXR* and *TGR5* mRNA (**A**) and FXR protein (**B**) in response to 1 μg/mL LPS with or without 1 mM UDCA. **C** The FXR expression after FXR siRNA interference. **D** FXR knockdown on phosphorylation of p65 NF-κB. Data are presented as means ± SEM. *P < 0.05, versus the control group, ^#^*P* < 0.05, versus LPS, ^&^*P* < 0.05, versus the LPS+UDCA group. **E** the mRNA expressions of *IL-1β* and *TNF-α* in response to 1 μg/mL LPS, 1 mM UDCA and/or FXR siRNA. Data are presented as means ± SEM. *P < 0.05, **P < 0.01, *** *P* < 0.001

FXR and TGR5 are two known receptors for BAs [45]. To study the involvement of both receptors in UDCA-mediated anti-inflammatory response, the mRNA expressions of *FXR* and *TGR5* were first examined. *TGR5* mRNA was not altered by UDCA in J774A.1 macrophage (data not shown), and a modest induction occurred at 6 and 12 h, but not 24 h, in the presence of LPS (Fig. 11A). On the other hand, *FXR* was induced by UDCA in J774A.1 cells in the presence (Fig. 11A) or absence of LPS (data not shown) for 6, 12, and 24 h. Moreover, UDCA enhanced the protein expression of FXR in J774A.1 macrophage with or without LPS (Fig. 11B). To further examine whether FXR is required to suppress UDCA-mediated NF-κB activation and inflammatory cytokine gene expression, RNA interference was employed. After FXR siRNA interference, the FXR expression was significantly downregulated (Fig. 11C). As expected, LPS-induced phosphorylation of NF-κB p65 was suppressed by UDCA; however, FXR knockdown using an FXR-specific siRNA partially abolished such a suppressive effect (Fig. 11D). Furthermore, FXR knockdown also partially reversed UDAC-mediated suppression of IL-1β expression and completely restored the expression of TNF-α (Fig. 11E). Taken together, these observations suggest that FXR is involved in UDCA-mediated suppression of LPS-induced NF-κB activation and proinflammatory cytokine production. The involvement of TGR5 in the anti-inflammatory effect of UDCA remains to be investigated, although it is likely to play a lesser role.

## Discussion

Infants with IUGR commonly exhibit feeding intolerance [7, 46, 47] and suffer from a higher risk for neonatal intestinal diseases such as necrotizing enterocolitis [48]. Previous studies in the piglet model showed increased levels of proinflammatory cytokines (IL-1β and TNF-α) in the colon of LBW animals [10–12]. However, the underlying mechanism remains elusive. Given a significant difference in the intestinal microbiome and metabolome between LBW and NBW piglets [22, 23] and a known link between microbial BA metabolism and IBD [20], we have examined the difference in the intestinal BA profile between LBW and NBW piglets through targeted profiling. We have further established a causal relationship between the intestinal microbiota and the BA profile and intestinal inflammation through FMT. Additionally, we have found that administration of UDCA, a BA that is highly diminished in LBW piglets, could directly alleviate intestinal inflammation in both LBW piglets, LBW-FMT mice, and a mouse model of colitis. Moreover, we have revealed the molecular and cellular mechanisms by which UDCA suppresses inflammation and demonstrated that macrophages are required for UDCA-mediated suppression of intestinal inflammation. UDCA exerts an anti-inflammatory effect in the intestinal tract by inducing M2 polarization of macrophages through FXR and inhibition of NF-κB. These findings have shed light on the involvement of BAs in LBW-associated intestinal abnormalities, suggesting the potential of targeting microbial BA metabolism for improving intestinal health and postnatal maldevelopment of LBW infants.

Early colonization of microbes in the neonatal gut plays an important role in regulating intestinal health. Compared with NBW piglets, the microbial α-diversity was higher in LBW piglets, which is inconsistent with the results of our previous study that the LBW piglets do not affect fecal microbial diversity at any age [22]. These differences may be caused by different breeds (Landrace × Yorkshire *vs* Duroc × (Landrace × Large White)), different sampling time points (day 3 after birth *vs* day 2 after birth), and different feces sampling methods (slaughter for rectal sample collection *vs* fresh feces sample collected by a sterile cotton swab) in the present study. FMT can provide new intuitive evidence for the mechanisms of gut microbiota-related diseases [49]. After FMT, despite that the recipient mice did not harbor a microbial community equal to the LBW and NBW donor newborn piglet, our results presented the different colonization of BA metabolism-related microbiome between LBW-FMT and NBW-FMT mice, reflected by the differences in intestinal BA-metabolizing enzyme (7α-HSDH and 7β-HSDH) and intestinal BA profile. This phenomenon of lower similarity between the donor and the recipient commonly happened in other publications involving FMT experiments [50–52]. It might be explained in the present study as although the changes in microbiota composition are not similar between the donor and the recipient, it may change the microbiome by FMT, especially the microbial function, which needs further research to confirmation.

BAs are increasingly appreciated to be critically involved in the regulation of inflammation through interacting with both microbiota and host receptors [53]. UDCA is a secondary BA and the bacterial 7β-OH epimer of the primary chenodeoxycholic acid (CDCA). Two key enzymes (7α-HSDH and 7β-HSDH) that are involved in the biosynthesis of UDCA from CDCA are encoded by several intestinal bacteria such as *Clostridium*, *Eubacterium*, *Bacteroides*, *E. coli*, and *Eggerthella lenta* [54, 55]. Among all differentially abundant BAs, UDCA was chosen for subsequent investigations in this study because (1) UDCA was the most differentially diminished BA in LBW piglets with the highest VIP value and the lowest *P*-value; (2) UDCA was significantly decreased in the colonic digesta of mice following FMT of LBW piglets; (3) Both *7α-HSDH* and *7β-HSDH* gene copy numbers were significantly reduced in the intestinal tract of both LBW piglets and LBW-FMT mice; (4) Relative abundances of *7α-HSDH-* and *7β-HSDH*-producing bacteria such as *Escherichia-Shigella* and *Clostridium sensu stricto* 2 in the feces of LBW piglets as well as *Bacteroides* and *Eggerthella* in the colon of LBW-FMT mice were significantly decreased; (5) A strong negative correlation was observed between the concentration of UDCA and the *7α-HSDH*/*7β-HSDH* gene copy number in both LBW piglets and LBW-FMT mice; (6) UDCA is the only BA have been used therapeutically for the treatment of a range of cholestatic disorders such as biliary atresia [56]; and (7) UDCA is known to be anti-inflammatory [57–59]. We have found that UDCA biosynthesis pathways are severely impaired in LBW and could potentially be utilized for LBW intervention. We have demonstrated that UDCA administration alleviates colonic inflammation in both LBW piglets, LBW-FMT mice, and a mouse model of colitis induced by DSS, consistent with several recent studies showing attenuation of DSS-induced colitis in mice by UDCA [57–59]. In fact, UDCA administration has also been shown to improve liver function in very low birth weight (VLBW) infants with total parenteral nutrition-associated cholestasis [60, 61]. In addition, UDCA was evidenced to block bacterial growth and invasion processes, mediating intestinal homeostasis by improving hindgut microflora structure and short-chain fatty acid production during extended-spectrum β-lactamase-producing *E. coli* infection in sepsis and colitis in neonatal mouse models [62].

Mechanistically, we have demonstrated for the first time that, at the cellular level, UDCA amelioration of intestinal inflammation is macrophage-dependent and achieved through preferential polarization of macrophages from the proinflammatory M1 phenotype toward the anti-inflammatory M2 phenotype [63], in agreement with recent studies showing a shift from the M1 to M2 macrophages in the alleviation of colitis [43, 64]. UDCA-induced M2 macrophage polarization was also shown in the adipose tissue of ob/ob mice [65]. In addition, similarly to our studies, UDCA was found to prevent colonic inflammation in TNBS- and DSS-treated rats or mice, a model of intestinal inflammation distinct from that used in the current studies [58, 66]. Thus, UDCA can prevent both the elevated cytokine levels and increased epithelial permeability associated with intestinal inflammation, suggesting it should be of therapeutic benefit in patients with IBD.

A growing body of evidence suggests that FXR is critical for mucosal homeostasis and generally suppressed during chronic intestinal inflammation [53]. BA-dependent FXR activation has been reported to suppress NF-κB activation by preventing nuclear co-receptor clearance from NF-κB-binding sites in the *TNF* and *IL-1β* loci [67, 68]. At the molecular level, we have shown that FXR is involved in UDCA-mediated suppression of NF-κB activation and inflammatory cytokine production both in vivo and in vitro. Consistently, FXR activation in mice with colitis shows reduced intestinal inflammation [69–71]. Conversely, FXR deficiency leads to heightened mucosal inflammation in mouse models of colitis [69]. Taken together, our results have suggested that UDCA reduces the inflammatory effects of macrophages via inhibiting NF-κB activation by interacting with FXR.

It is worth noting that, in addition to FXR, BAs are ligands to several other nuclear receptors such as pregnane X receptor (PXR), glucocorticoid receptor (GR), and vitamin D receptor (VDR) as well as a cell surface receptor (TGR5) [72]. Some of these receptors are also likely to be involved in UDCA-mediated anti-inflammatory response because FXR knockdown fails to completely suppress NF-κB activation and inflammatory cytokine production in response to UDCA, although the incomplete suppression could also be due to a relatively low gene knockdown efficiency in macrophages. Nevertheless, the possible involvement of other BA receptors (e.g., PXR, GR, VDR) warrants further investigation.

Although UDCA was chosen in this study and subsequently confirmed to alleviate intestinal inflammation upon oral administration into LBW piglets, other differentially abundant BAs are likely to play a role as well. It will be important to examine the efficacy of other BAs or a combination of BAs in mitigating LBW-associated metabolic and immunological abnormalities. Secondly, because 7α-HSDH and 7β-HSDH, two major enzymes involved in the biosynthesis of UDCA, are significantly reduced in LBW piglets, it will be interesting to investigate the potential of transferring 7α-HSDH- and 7β-HSDH-positive bacteria for the treatment of postnatal maldevelopment of LBW infants. Thirdly, because we have shown the intestinal microbiota is responsible for much of the intestinal health issues associated with LBW piglets, it is possible to achieve a positive outcome by considering the FMT of normal individuals to LBW infants. Fourthly, it does not fully explain the mechanism of UDCA improving LBW-induced intestinal inflammation through macrophage polarization using an in vitro macrophage cell model. Further macrophage adoptive transfer in vivo is still needed.

## Conclusion

In summary, we have revealed for the first time that LBW piglets produce a significantly lower amount of UDCA and harbor less 7α-HSDH- and 7β-HSDH-positive bacteria than their normal counterparts. Oral supplementation of UDCA significantly improves the intestinal health of LBW piglets. We have further demonstrated that UDCA alleviates intestinal inflammation by regulating macrophage polarization, engaging FXR, and suppressing NF-kB activation. This study provides a direct link between LBW-associated intestinal abnormalities and altered microbiota-mediated BA metabolism. Taken together, we have not only revealed the mechanisms of LBW-associated intestinal disorder, but also proposed several independent, but related, intervention strategies for the treatment of LBW-associated postnatal abnormalities.

## Supplementary Information

**Additional file 1: Supplemental Table S1.** Scoring standards for the disease activity index (DAI)^1,2^. **Supplemental Table S2.** Parameters and criteria of histological damage evaluation^1,2^. **Supplemental Table S3.** Primer pairs used in this experiment^1^. **Supplemental Figure S1.** Fecal bile acids (BAs) profiles of low birth weight (LBW) and normal birth weight (NBW) piglets 2 days after birth (*n* = 10). **Supplemental Figure S2.** Verification of depletion of the intestinal microbiota in mice (*n* = 8). **Supplemental Figure S3.** Microbial structure of NBW and LBW donor piglets and NBW-FMT and LBW-FMT recipient mice. **Supplemental Figure S4.** Microbial composition of NBW and LBW donor piglets and NBW-FMT and LBW-FMT recipient mice. **Supplemental Figure S5.** Colonic BA profiles of mice transplanted with the feces of LBW or NBW piglets (*n* = 8). **Supplemental Figure S6.** Effect of UDCA on inflammatory cytokines gene expression and macrophage polarization in J774A.1 cell.

## Data Availability

All sequence reads were deposited into the NCBI Sequence Read Archive (SRA) database under accession number PRJNA646844 for the feces of piglets and PRJNA714049 for the colonic digesta of mice after FMT. All RNA sequencing data were deposited in NCBI’s Gene Expression Omnibus (GEO) under the accession number GSE174489.
