# Supplementary material for: Gut microbiota-derived ursodeoxycholic acid alleviates low birth weight-induced colonic inflammation by enhancing M2 macrophage polarization
Source: Microbiome. 2023 Jan 31;11:19. doi: 10.1186/s40168-022-01458-x (PMC9887892; doi:10.1186/s40168-022-01458-x)
Supplement: Supplementary file 2 — Additional file 1: Supplemental Table S1. Scoring standards for the disease activity index (DAI)1,2. Supplemental Table S2. Parameters and criteria of histological damage evaluation1,2. Supplemental Table S3. Primer pairs used in this experiment1. Supplemental Figure S1. Fecal bile acids (BAs) profiles of low birth weight (LBW) and normal birth weight (NBW) piglets 2 days after birth (n = 10). Supplemental Figure S2. Verification of depletion of the intestinal microbiota in mice (n = 8). Supplemental Figure S3. Microbial structure of NBW and LBW donor piglets and NBW-FMT and LBW-FMT recipient mice. Supplemental Figure S4. Microbial composition of NBW and LBW donor piglets and NBW-FMT and LBW-FMT recipient mice. Supplemental Figure S5. Colonic BA profiles of mice transplanted with the feces of LBW or NBW piglets (n = 8). Supplemental Figure S6. Effect of UDCA on inflammatory cytokines gene expression and macrophage polarization in J774A.1 cell. [file 40168_2022_1458_MOESM1_ESM.docx]

**Supplemental Tables and Figures**

**Supplemental Table S1. Scoring standards for the disease activity index (DAI)^1,2^**

| Score | Weight loss (%) | Stool consistency | Fecal blood content |
| --- | --- | --- | --- |
| 0 | None | Normal | Normal |
| 1 | 0-10 |  |  |
| 2 | 11-15 | Loose stool | Occult blood |
| 3 | 16-20 |  |  |
| 4 | >20 | Diarrhea | Hemorrhage/Gross bleeding |

^1^ The DAI score was defined as the sum of scores from weight loss (%), stool consistency, and fecal blood content.

^2^ Adapted from Park et al.

**Supplemental Table S2. Parameters and criteria of histological damage evaluation^1,2^**

| Parameters | Score | Histological features |
| --- | --- | --- |
|  | 0 | No change |
| 1. Loss of epithelial surface | 1 | Localized and mild |
| 1. Destruction of crypt | 2 | Localized and moderate |
| 1. Infiltration of inflammatory cells | 3 | Localized and severe |
|  | 4 | Extensive and moderate |
|  | 5 | Extensive and severe |

^1^ Histological score was the sum of scoring from parameters (1), (2), and (3).

^2^ Adapted from Wang *et al*. with modifications.

**Supplemental Table S3. Primer pairs used in this experiment^1^**

| Target gene | Forward primer (5'-3') | Reverse primer (5'-3') | Annealing temp. (°C) | | References | |
| --- | --- | --- | --- | --- | --- | --- |
| **For host genes of pig** | | | | | | |
| OCLN | CAGCCTCGGTACAGCAGCAAT | ATAGTGGTCAGGGTCCGTCCTC | 60 | | Huang *et al*., 2020 | |
| ZO-1 | CGGAACTATGACCATCGCCTAC | CTTCGGGATGTTGTCTGGAGTC | 60 | | Huang *et al*., 2020 | |
| CLDN1 | AGCTGTGCATGGCCTCTTGT | CCAATGTCAATGGCAACACCCT | 60 | | Huang *et al*., 2020 | |
| MUC1 | AATGGCTCCTCGGTGCTACCTA | TGACTTGGCACTGAAGGCTGAG | 60 | | Huang *et al*., 2020 | |
| MUC2 | TGCTGACGAGTGGTTGGTGAATG | GATGAGGTGGCAGACAGGAGACA | 60 | | Huang *et al*., 2020 | |
| IL-1β | CTCGCAGCAGCACATCAACAAG | GGAAGGTCCACGGGAAAGACAC | 60 | | Huang *et al*., 2020 | |
| TNF-α | TGGGAGTAGACAAGGTACAACCC | CATCTTCTCAAAATTCGAGTGACAA | 60 | | Huang *et al*., 2020 | |
| FXR | TATGAACTCAGGCGAATGCCTGCT | ATCCAGATGCTCTGTCTCCGCAAA | 60 | | Radtke *et al*., 2014 | |
| TGR5 | CCATGCACCCCTGTTGCT | GGTGCTGTTGGGTGTCATCTT | 60 | Ipharraguerre *et al*., 2013 | |  |
| SHP | GCCTACCTGAAAGGGACCAT | CAACGGGTGTCAAGCCTTTA | 60 | Vlaardingerbroek *et al*., 2014 | |  |
| FGF19 | AAGATGCAAGGGCAGACTCA | AGATGGTGTTTCTTGGACCAGT | 60 | Vlaardingerbroek *et al*., 2014 | |  |
| *β-actin* | ATGCTTCTAGACGGACTGCG | GTTTCAGGAGGCTGGCATGA | 60 | | Lin *et al*., 2014 | |
| *GAPDH* | TTTGCGTCAGTGTCATCG | TGCTCTGCCTTGGGTAAT | 60 | | Fang *et al*., 2014 | |
| **For host genes of mouse** | | | | | | |
| ZO-1 | CGGAACTATGACCATCGCCTAC | CTTCGGGATGTTGTCTGGAGTC | 60 | | Huang *et al*., 2019 | |
| OCLD | CAGCCTCGGTACAGCAGCAAT | ATAGTGGTCAGGGTCCGTCCTC | 60 | | Huang *et al*., 2019 | |
| IL-1β | CTCGCAGCAGCACATCAACAAG | GGAAGGTCCACGGGAAAGACAC | 60 | | Huang *et al*., 2019 | |
| TNF-α | TGGGAGTAGACAAGGTACAACCC | CATCTTCTCAAAATTCGAGTGACAA | 60 | | Huang *et al*., 2019 | |
| IL-6 | ACCACGGCCTTCCCTACTT | CACAACTCTTTTCTCATTTCCAC | 60 | | Huang *et al*., 2019 | |
| IL-10 | CCCTTTGCTATGGTGTCCTT | TGGTTTCTCTTCCCAAGACC | 60 | This study; NM_010548.2 | |  |
| MUC2 | TGCTGACGAGTGGTTGGTGAATG | GATGAGGTGGCAGACAGGAGACA | 60 | | Huang *et al*., 2019 | |
| FXR | TGGGCTCCGAATCCTCTTAGA | TGGTCCTCAAATAAGATCCTTGG | 60 | | Sun *et al*., 2018 | |
| TGR5 | CCTGGCAAGCCTCATCGTC | AGCAGCCCGGCTAGTAGTAG | 60 | | Sun *et al*., 2018 | |
| SHP | CAGGTCGTCCGACTATTCTG | ACTTCACACAGTGCCCAGTG | 60 | This study; NM_011850.3 | |  |
| FGF15 | ACGGGCTGATTCGCTACTC | TGTAGCCTAAACAGTCCATTTCCT | 60 | This study; NM_008003.2 | |  |
| β-actin | TGGAATCCTGTGGCATCCATGAAAC | TAAAACGCAGCTCAGTAACAGTCCG | 60 | | Huang *et al*., 2019 | |
| GAPDH | AACTTTGGCATTGTGGAAGG | ACACATTGGGGGTAGGAACA | 60 | | Wang et al., 2020 | |
| **For bacteria** |  |  |  | |  | |
| Total bacteria | GTGSTGCAYGGYYGTCGTCA | ACGTCRTCCMCNCCTTCCTC | 60 | | Maeda *et al*., 2003 | |
| *baiJ* | TCAGGACGTGGAGGCGATCCA | TACRTGATACTGGTAGCTCCA | 60 | | Yoshimoto *et al*., 2013 | |
| *bsh* | ATGGGCGGACTAGGATTACC | TGCCACTCTCTGTCTGCATC | 54 | | Duary *et al*., 2012 | |
| *7α-HSDH* | GGGTATTGTGTATCAAAAGCTGCGG | TCCGTTGCTATAAGCCCAGGTAAGA | 60 | | This study; MH743112.1 | |
| *7β-HSDH* | GTCGTAAAAGCAGACTTTTCGCTGC | TTGATCATTGCCTCATGCTTTTCC | 60 | | This study; KF052988.1 | |

^1^ZO-1, zonula occludens-1; ZO-2, zonula occludens-2; MUC, Mucin; CLDN, claudin; OCLN, Occludin; IL-6, interleukin-6; TNF-α, tumor necrosis factor-α; GAPDH, glyceraldehyde-3-phosphate dehydrogenase; FXR, farnesoid X receptor; TGR5, G-coupled protein receptor; SHP, small heterodimer partner; FGF15/19, fibroblast growth factor 15/19; *baiJ*, genes encoding bile salt 7α-dehydroxylase; *bsh*, genes encoding bile salt hydrolase; 7α-HSDH, genes encoding 7α-hydroxysteroid dehydrogenase; 7β-HSDH, genes encoding 7β-hydroxysteroid dehydrogenase.


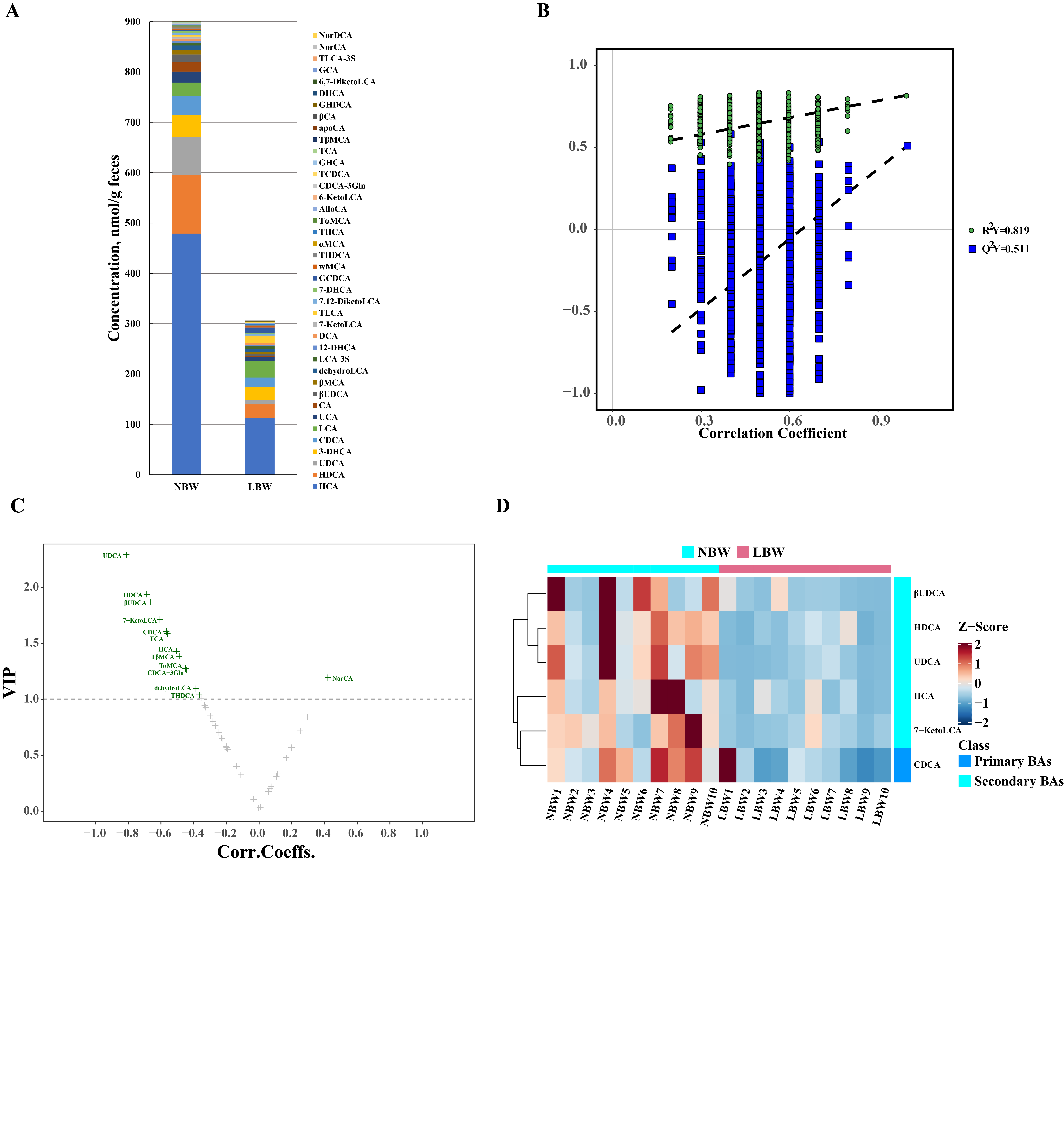


**Supplemental Figure S1. Fecal bile acids (BAs) profiles of low birth weight (LBW) and normal birth weight (NBW) piglets 2 days after birth (n = 10)**. (**A**) Relative abundances of different BAs in the feces of NBW and LBW piglets. (**B**) Validation plot of the OPLS-DA model of the fecal BAs profiles. (**C**) Volcano plot of variable importance in projection (VIP) of different BAs based on the OPLS-DA model. (**D**) Z-Scores of six most differentially abundant fecal BAs in individual piglets.


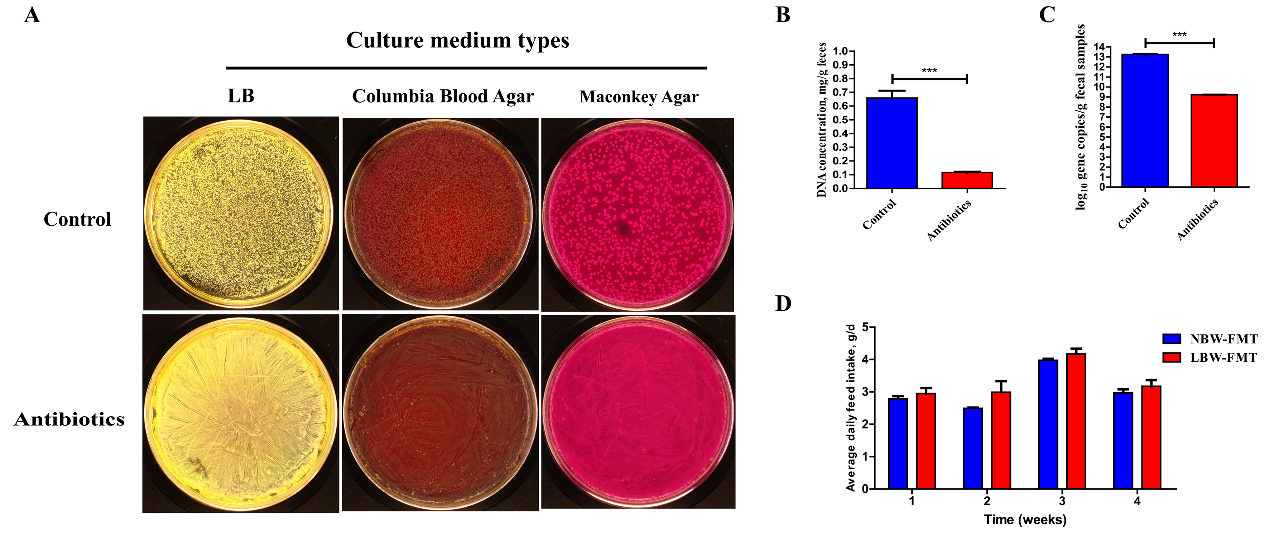


**Supplemental Figure S2. Verification of depletion of the intestinal microbiota in mice (n = 8).** (**A**) Fecal microbiota culture on LB, blood agar, and McConkey agar after two weeks of treatment with a cocktail of antibiotics. (**B**) The concentration of fecal microbial DNA in mice after two weeks of antibiotic treatment. (**C**) The genome copies the number of total bacteria in the feces of mice after two weeks of antibiotic treatment. (**D**) The average daily feed intake of mice during transplanted with the feces of NBW and LBW piglets. Data are presented as means ± SEM. *** *P* < 0.001. LBW-FMT, mice transplanted with the feces of LBW piglets; NBW-FMT, mice transplanted with the feces of NBW piglets.





**Supplemental Figure S3. Microbial** **structure of NBW and LBW donor piglets and NBW-FMT and LBW-FMT recipient mice.** (**A**) Rarefaction curves. (**B**) The α-diversity of the fecal microbiota of NBW and LBW donor piglets. (**C**) The α-diversity of the colonic digesta microbiota of NBW-FMT and LBW-FMT recipient mice. (**D**) Principal coordinates analysis (PCoA) plot of the microbiomes between the donor piglets and the recipient mice, and (**E**) the fecal microbiomes of LBW and NBW piglets or (**F**) the colonic microbiomes of NBW-FMT and LBW-FMT mice based on the weighted unifrac distance. (**G**) PCoA plot of the microbiomes between the donor piglets and the recipient mice, and (**H**) the fecal microbiomes of LBW and NBW piglets or (**I**) the colonic microbiomes of NBW-FMT and LBW-FMT mice based on the unweighted unifrac distance. Data are presented as means ± SEM in panels **B** and **C**. **P* < 0.05. LBW-FMT, mice transplanted with the feces of LBW piglets; NBW-FMT, mice transplanted with the feces of NBW piglets.





**Supplemental Figure S4. Microbial composition of NBW and LBW donor piglets and NBW-FMT and LBW-FMT recipient mice.** (**A**) Relative abundance of microbial composition in fecal samples of NBW and LBW donor piglets and colonic digesta samples of NBW-FMT and LBW-FMT recipient mice at the phylum level. (**B**) Relative abundance of microbial composition in fecal samples of NBW and LBW donor piglets and colonic digesta samples of NBW-FMT and LBW-FMT recipient mice at the genus level. (**C**) Differential enrichment of bacteria in fecal samples of NBW and LBW donor piglets at the phylum level. (**D**) Differential enrichment of bacteria in colonic digesta samples of NBW-FMT and LBW-FMT recipient mice at the phylum level. Data are presented as means ± SEM. **q* < 0.05, ***q* < 0.01. LBW-FMT, mice transplanted with the feces of LBW piglets; NBW-FMT, mice transplanted with the feces of NBW piglets.


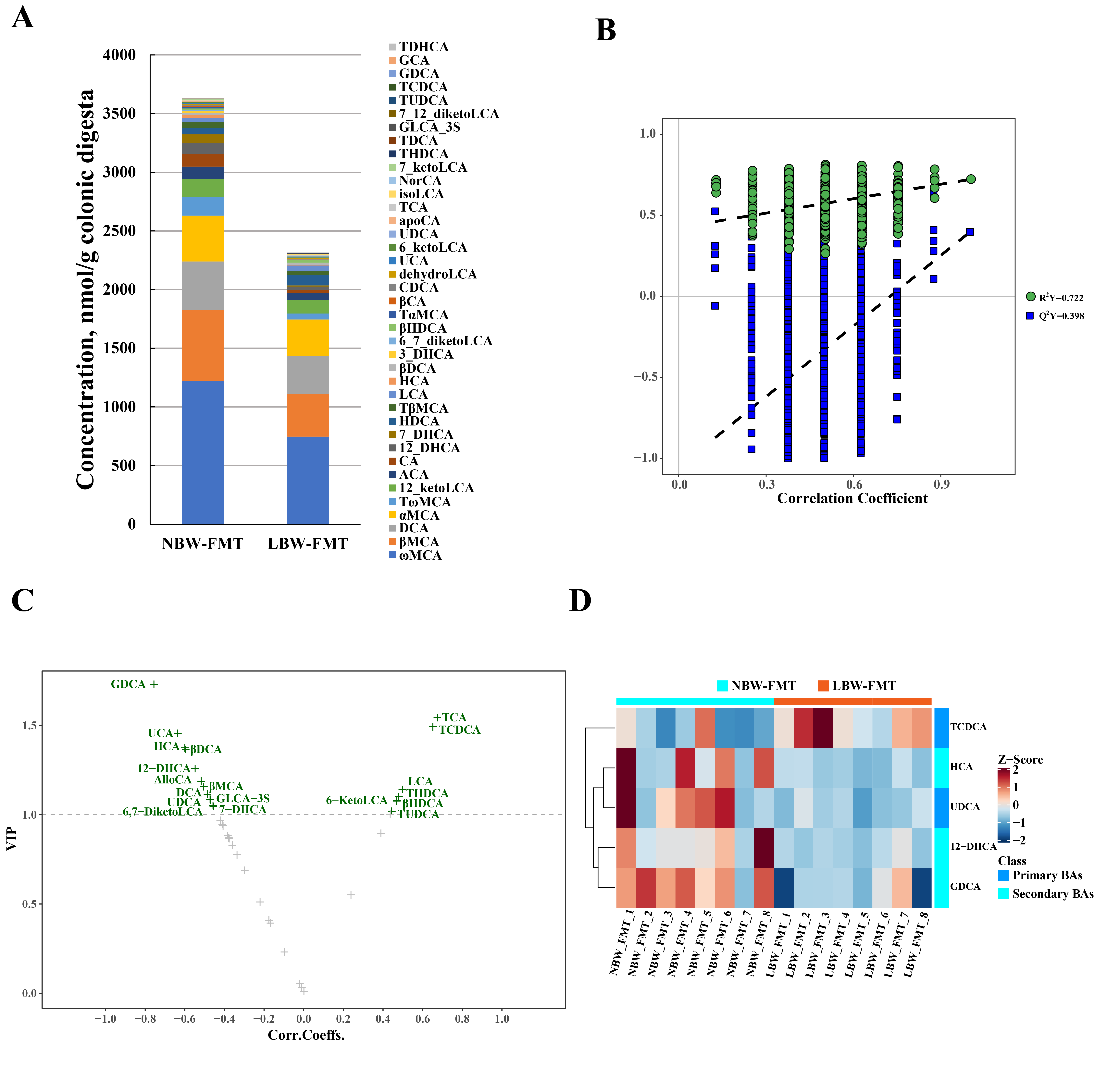
**Supplemental Figure S5. Colonic BAs profiles of mice transplanted with the feces of LBW or NBW piglets (n = 8)**. (**A**) Relative abundances of different BAs in the colon of mice transplanted with the feces of NBW and LBW piglets. (**B**) Validation plot of the OPLS-DA model of the fecal BAs profiles. (**C**) Volcano plot of variable importance in projection (VIP) of different BAs based on the OPLS-DA model. (**D**) Z-Scores of five highly differentially abundant colonic BAs in individual animals. LBW-FMT, mice transplanted with the feces of LBW piglets; NBW-FMT, mice transplanted with the feces of NBW piglets.


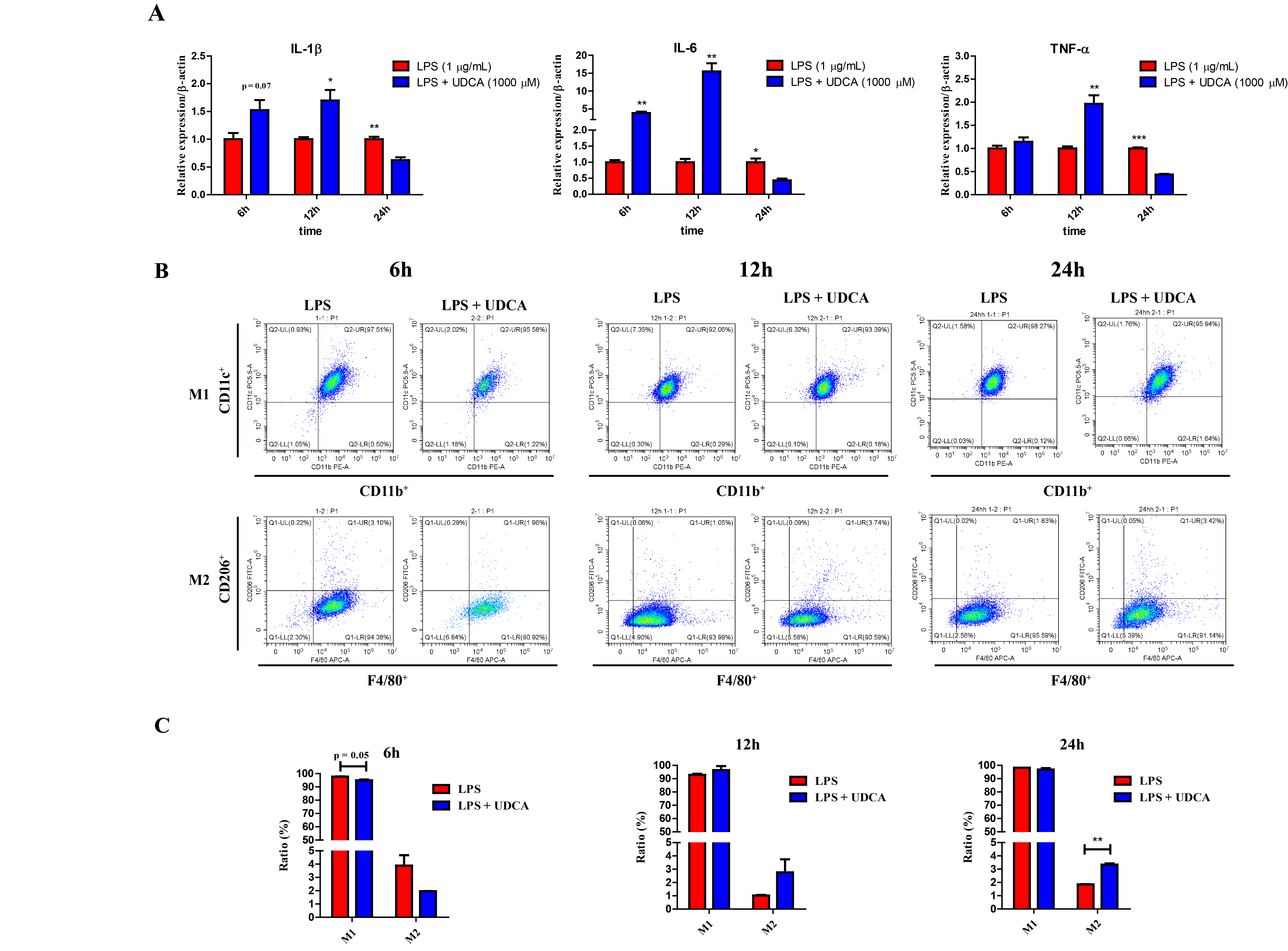
**Supplemental Figure S6. Effect of UDCA on inflammatory cytokines gene expression and macrophage polarization in J774A.1 cell**. (**A**) The mRNA expressions of three pro-inflammatory cytokine genes in J774A.1 cell in response to 1 μg/mL LPS with or without 1 mM UDCA. (**B**) The representative flow cytometry plots, (**C**) as well as the prevalence of M1 macrophages (CD11b^+^CD11c^+^) and M2 macrophages (F4/80^+^CD206^+^) in the J774A.1 cell in response to UDCA. Data are presented as means ± SEM. **P* < 0.05, ***P* < 0.01, ****P* < 0.001.
